# Graphene-Ag Hybrids on Laser-Textured Si Surface for SERS Detection

**DOI:** 10.3390/s17071462

**Published:** 2017-06-22

**Authors:** Chentao Zhang, Kun Lin, Yuanqing Huang, Jianhuan Zhang

**Affiliations:** 1 Department of Instrumental and Electrical Engineering, Xiamen University, Xiamen 361005, China; zctyyy@163.com (C.Z.); KunLin429@foxmail.com (K.L.); yqhuang@xmu.edu.cn (Y.H.); 2 Xiamen Key Laboratory of Optoelectronic Transducer Technology, Xiamen 361005, China; 3 Fujian Key Laboratory of Universities and Colleges for Transducer Technology, Xiamen 361005, China

**Keywords:** surface-enhanced Raman scattering, graphene-silver plasmonic hybrids, laser ablation

## Abstract

Surface-enhanced Raman scattering (SERS) has been extensively investigated as an effective approach for trace species detection. Silver nanostructures are high-sensitivity SERS substrates in common use, but their poor chemical stability impedes practical applications. Herein, a stable and sensitive SERS substrate based on the hybrid structures of graphene/silver film/laser-textured Si (G/Ag/LTSi) was developed, and a simple, rapid, and low-cost fabrication approach was explored. Abundant nanoparticles were directly created and deposited on the Si surface via laser ablation. These aggregated nanoparticles functioned as hotspots after a 30 nm Ag film coating. A monolayer graphene was transferred to the Ag film surface to prevent the Ag from oxidation. The SERS behavior was investigated by detecting R6G and 4-MBT molecules. The experimental results indicate that the maximum enhancement factor achieved by the G/Ag/LTSi substrate is over 10^7^ and less than 23% SERS signals lost when the substrate was exposed to ambient conditions for 50 days. The covering graphene layer played crucial roles in both the Raman signals enhancement and the Ag nanostructure protection. The stable and sensitive SERS performance of G/Ag/LTSi substrate evince that the present strategy is a useful and convenient route to fabricate large-area graphene-silver plasmonic hybrids for SERS applications.

## 1. Introduction

Trace species detection is important in our daily life, such as in the fields of food safety, environmental monitoring, and disease diagnosis. SERS is an attractive analytical technique for label-free detection and identification of chemical and biological species at trace concentrations owing to its outstanding capability for tremendously boosting the pristine Raman signals [1,2,3,4]. SERS has attracted considerable attention since it was first observed on a roughened silver electrode in 1974 [5]. It has been widely accepted that the enormous enhancement of Raman signals is mainly ascribed to the combination of electromagnetic mechanism (EM) and chemical mechanism (CM) [2]. EM, a major contributor to enhanced Raman signals, originates from the remarkably magnified electromagnetic field induced by a light-excited surface plasmon resonance [6]. The strong electromagnetic fields are usually located at the sharp corners of metal nanostructures and the nano-gaps between these nanostructures, known as “hotspots” [7]. The enhancement induced by the EM is proportional to |E|^4^, where E is the intensity of the electromagnetic field. The CM coexists with the EM for a typical SERS system, which derives from the charge transfer between the analytical molecules and SERS substrates with an enhancement factor (EF) of around 10–100.

Gold, silver (Ag), and copper nanostructures or roughened surfaces are commonly used SERS substrates [8]. Among these metal materials, Ag has been demonstrated to have the highest EF, owing to its strong plasmonic enhancement effect and low loss at optical frequencies [9,10]. However, Ag is prone to oxidation by atmospheric oxygen and water vapor when exposed to ambient conditions, which causes significant changes to chemical and plasmonic properties and restricts the long-term usage of Ag-based SERS substrates [9,11]. Many strategies have been proposed to improve the chemical stability of Ag nanostructures. Coating a stable protective layer or shell made up of inert materials on Ag nanostructures is the most common approach [12,13,14]. Inert materials—such as SiO_2_ [12], TiO_2_ [13], Al_2_O_3_ [12], and polymer [14]—have been reported to serve as protective layer/shell. Surface plasmon resonance is a near-field interaction by nature. Electromagnetic field enhancement decays exponentially away from the Ag surface [15]. Therefore, an inert, ultrathin, and pinhole-free isolating layer/shell is essential for the full protection of Ag nanostructures without compromising the Raman enhancement. However, preparing such a protective layer/shell is rather demanding and challenging, requiring meticulous treatment of the substrates, strict reaction conditions, and long reaction time [16].

Graphene is a single layer of sp^2^-bonded carbon atoms densely packed into two-dimensional honeycomb crystal lattices with fascinating properties, such as atomic thickness, high optical transparency, chemical inertness, biological compatibility, excellent flexibility, and impenetrability to gas molecules. Such merits endow graphene as an ideal material for the protective layer/shell in SERS applications. Graphene can be easily obtained by chemical vapor deposition or mechanical exfoliation from HOPG. Moreover, it has been demonstrated to serve as an effective substrate for Raman enhancement with an EF of 2–17 attributed to the CM [17]. Several hybrid SERS substrates composed of graphene and metal nanostructures have been developed recently [10,18,19,20,21,22], which retain the electromagnetic enhancement derived from metal nanostructures and simultaneously employ the unique properties of graphene. For these hybrid SERS substrates, apart from the initial functions as a protective layer [10] and an additional chemical enhancer [19], graphene also serves as a molecule enricher [20], a spacer layer [21], and a fluorescence quencher [22], which makes graphene-metal hybrids become a stable, high-sensitive, multifunctional, and promising platform for SERS detections.

Laser ablation has initiated a facile route to directly fabricate micro/nanostructures on solid substrates with the merits of high speed, low cost, and high throughput [23]. It provides a good solution for the fabrication of SERS substrates over a large area [24,25,26,27,28]. Herein, motivated by the above-mentioned advantages of graphene and laser ablation, we provide a rapid, simple, and low-cost approach to fabricate sensitive and stable graphene-silver plasmonic hybrids for SERS applications. This substrate is combined of single-layer graphene, Ag film and laser-textured Si surface, forming the hybrid graphene/Ag/laser-textured Si (G/Ag/LTSi) structure. Using rhodamine 6G (R6G) and 4-methylbenzenethiol (4-MBT) molecules as probe molecules, the G/Ag/LTSi substrate exhibits stable and sensitive SERS performance.

## 2. Experimental Section

**Figure 1:**
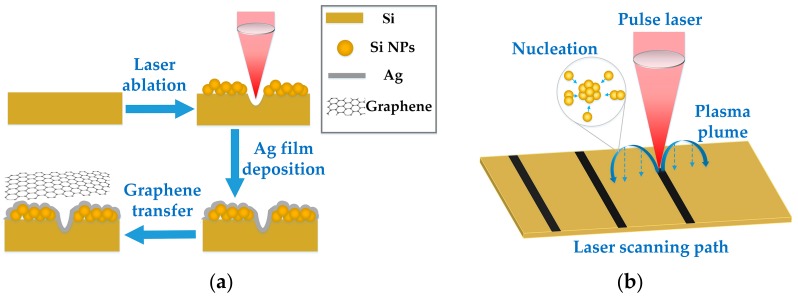
Schematic diagram of (**a**) the fabrication process of G/Ag/LTSi SERS substrates and (**b**) laser ablation of Si surface for nanoparticles deposition.

Figure 1a illustrates the fabrication procedure of G/Ag/LTSi SERS substrates. Nanoparticles were generated directly on the Si surface by laser ablation. Then an Ag thin film was deposited on the laser-textured Si surface to form Ag nanostructures. Finally, monolayer graphene was transferred to the surface of Ag nanostructures to function as a protective layer and an additional enhancer to improve the SERS behavior.

### 2.1. Laser Ablation of Si Substrate

A fiber pulse laser with a wavelength (λ) of 1064 nm, pulse duration (τ) of 10 ns and pulse repetition rate (PRR) of 100 KHz was utilized to generate nanoparticles on the surface of n-type Si (100) substrate in ambient air, as illustrated in Figure 1b. The laser pulses were driven to scan along the Si surface by a laser galvo-scanner at a speed (v) of 100 mm/s and focused to ~20 μm by a F-Theta lens. Pulse laser ablation of Si substrate is a complicated process for the Si target materials removing and nanoparticles synthesis, involving laser–Si substrate interaction, laser–plasma interaction, and plasma–air molecule interaction [29,30]. The whole process can be divided into five steps, including: the formation of plasma species, the generation of shock wave, the expansion of plasma plume, the nucleation and growth of nanoparticles, as well as the transport and deposition of nanoparticles [30,31]. When the pulse laser with high enough fluence ablates the Si substrate, the absorbed high-intensity laser energy causes the breakdown of Si target material and the formation of plasma. In the area near the Si target, the gaseous energetic plasma species consisted of atoms, ions, clusters, and particulates spread out with high initial kinetic energy due to the shock wave. The strong interaction between the plasma species and the air molecules decreases the kinetic energy of plasma species gradually and induces their nucleation, which promotes the aggregation of plasma species to form nanoparticles. These synthesized nanoparticles gradually release the kinetic energy during transport and are deposited onto the Si surface due to the gravity force acting.

### 2.2. Ag Film Deposition

A 30 nm Ag film was deposited on the surface of laser-textured Si to form the hybrid Ag/LTSi substrate. The deposited Ag film functioned as Ag nanostructures for SERS detection. Ag film deposition was conducted by an Edwards Vacuum E-beam Evaporation System with a chamber pressure below 6 × 10^−6^ Torr and a coating rate at 0.2 nm/s.

### 2.3. Graphene Synthesis and Transfer

Large-area monolayer graphene was synthesized on a 25 μm thick copper foil (Alfa Aesar) in a tube furnace with CH_4_ as the carbon source by chemical vapor deposition (CVD). Firstly, the Cu foil was annealed for 10 min at 1030 °C, maintaining a H_2_ flow rate of 10 sccm. Then the gas mixture of CH_4_ (10 sccm) and H_2_ (20 sccm) was introduced into the quartz tube for 20 min to synthesize graphene. A total pressure of 500 mTorr was maintained in the tube during graphene growth. Finally, the Cu foil was cooled down to room temperature with flowing H_2_ at a flow rate of 20 sccm. Then the as-grown monolayer graphene was transferred onto the surface of Ag/LTSi substrate by wet chemical etching. Polymethylmethacrylate (PMMA) was firstly spin-coated on one side of the Cu foil. Next the Cu foil was etched by 0.1 M FeCl_3_ solution, leaving a graphene/PMMA film floating on the etchant. Afterward, this hybrid film was washed with deionized water to remove the residual FeCl_3_ and then collected by the as-prepared Ag/LTSi substrate. Finally, the substrate with the adsorbed graphene/PMMA layer was immersed into acetone to remove the PMMA layer and then washed by deionized water.

### 2.4. Characterization and SERS Measurement

The surface morphology of laser-textured Si substrate was observed by a confocal microscope (TCS SP5, Leica Microsystems Inc., Buffalo Grove, IL, USA) and a field emission scanning electron microscopy (FESEM, Zeiss Supra 55, Carl Zeiss AG, Feldbach, Switzerland). The atomic constitution of substrates was obtained by an energy dispersive spectroscopy (EDS, Inca X-Max 50, Oxford Instruments, Oxfordshire, UK) equipped on the FESEM. The SERS signals were collected by a laser confocal Raman spectrometer (LabRAM HR Evolution, Horiba Jobin Yvon, Palaiseau, France) equipped with a 50× microscope objective lens (NA = 0.75). When conducting SERS signals measurements, the employed laser power is 10 mW and laser excitation wavelength is 532 nm. The used integration time is less than two seconds to prevent a major heating effect induced by laser irradiation.

R6G and 4-MBT were utilized as probe molecules to test the SERS performances. In order to form a self-assembled monolayer of probe molecules on the surface of SERS substrate, the as-prepared SERS substrate were submerged inside the solution of probe molecules with a specific concentration for two hours and then rinsed in deionized water for 30 s, followed by nitrogen drying.

## 3. Results and Discussion

### 3.1. Characterization and SERS Behavior of the Ag/LTSi Substrate

**Figure 2:**
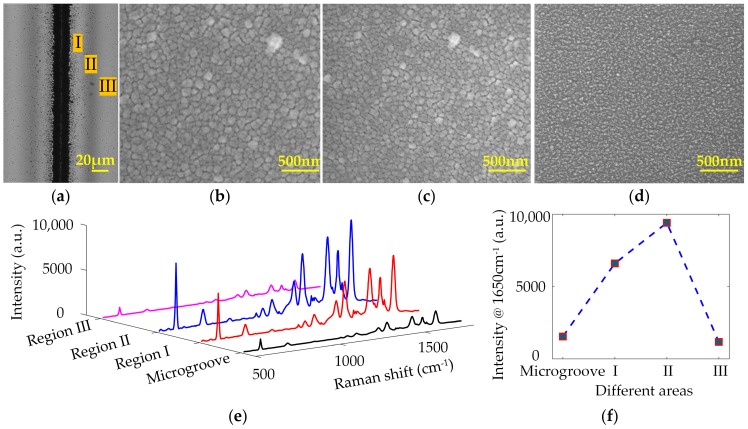
(**a**) Microscope image of the microgroove created by single-line laser ablation of Si surface (λ = 1024 nm, τ = 10 ns, PRR = 100 KHz, laser spot size = ~20 μm, v = 100 mm/s and laser fluence = 15 J/cm^2^); SEM images of regions marked with I (**b**), II (**c**), and III (**d**), in (**a**); (**e**) SERS spectra, and (**f**) SERS intensity at 1650 cm^−1^ of R6G on different regions of single-line laser-ablated Si surface with 30 nm Ag film coating.

Figure 2a depicts the microscope image of microgroove pattern on the Si surface fabricated by single-line laser ablation at a laser fluence of 15 J/cm^2^. The microgroove is the region irradiated directly by focal laser pulses, which cause the mass removal on Si surface. The width of microgroove is ~20 μm determined by the focal laser spot size. During laser ablation, different sizes of nanoparticles are generated and decorated on the microgroove pattern as well as the surrounding areas. The single-line laser ablation configures the Si surface into four geometry regions with different morphologies, including microgroove, Region I, Region II, and Region III. It can be observed that Regions I, II, III appear as various gray scales in the microscope image. That is ascribed to the different light absorption in these regions, suggesting the various morphology features of surface structures. Figure 2b–d show the SEM images of surface nanostructures in Regions I, II, and III, respectively. The sizes and distribution of nanoparticles are gradually changed with the distance away from the microgroove. Region I, the region near the microgroove (<20 μm), is decorated with large-size nanoparticles. In Region II, 20~40 μm away from the microgroove, nanoparticles with smaller size can be observed. Region III is the area far from the microgroove (40~80 μm), where few nanoparticles are found since most of them cannot be transported to such a long distance.

Since the Si substrate was fabricated in air environment, its surface was partially oxidized due to the high pressure and temperature induced by laser ablation. An EDX measurement was conducted to roughly investigate the change of Si surface’s constitution. The results show that the content of oxygen element on Si surface increases from nearly 0–11% after laser ablation at a laser fluence of 15 J/cm^2^. It should be noted that the oxidation of Si cannot affect its SERS behavior because the ablated Si surface is only used to provide the nanoscale surface structures for the formation of Ag nanostructures after Ag film deposition. This is also the reason that the Si substrate can be fabricated directly in ambient air without protection.

Laser ablation creates substantial nanostructures on Si surface by the aggregation of abundant nanoparticles. In order to test its SERS behavior, the ablated Si surface was deposited with a 30 nm Ag thin film by an electron beam evaporator. Then the substrate was immersed into 10^−2^ mM R6G solution for the formation of monolayer prober molecules via self-assembly. Figure 2e illustrates the SERS signals of R6G in each region. All the quintessential vibrational modes including 612, 774, 1360, 1509, 1574, 1599, and 1650 cm^−1^ can be observed and agree with those reported previously [18,32]. It should be noted that the SERS signals shown in Figure 2e are the surface enhanced resonance Raman scattering (SERRS) signals in nature. The excitation wavelength of the laser used is located in the neighborhood of R6G’s electronic transition frequency. Both enhancement from the surface plasmon resonance and the molecular resonance of R6G molecules contribute to the detected Raman signals and the former is the major contributor. Figure 2f shows the SERS intensity of Raman peak at 1650 cm^−1^ in each region. The microgroove region exhibits the limited Raman signals (~1109 counts at 1650 cm^−1^) since the microgroove with a depth of tens of microns confines the scattered Raman signals inside and causes them hardly to be detected. The Raman signals in Regions I and II are stronger, where plenty of nanoparticles are deposited on the Si surface, leading to the formation of substantial nanostructures and abundant nanogaps between the adjacent nanoparticles. These substantial nanostructures provide larger surface area for the absorption of probe molecules. More importantly, with Ag film deposition, Ag nanostructures are formed and plenty of hotspots are created by the aggregation of the Ag coated nanoparticles. It can be further observed that the SERS performance in Region II (~9396 counts at 1650 cm^−1^) is better than Region I (~6548 counts at 1650 cm^−1^). That is because that the smaller nanoparticles in Region II generate more hotspots to induce a more intense local electromagnetic field. Region III (~742 counts at 1650 cm^−1^) shows the weakest SERS signals as few nanoparticles can arrive this region to form nanostructures.

**Figure 3:**
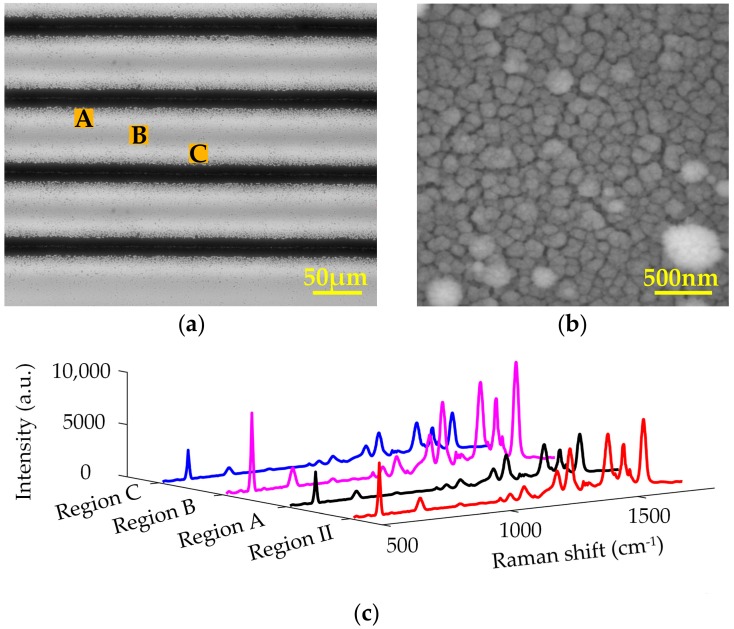
(**a**) Microscope image of the microgroove array created on Si surface by multi-line laser ablation; (**b**) SEM image of the region marked with B in (**a**); (**c**) SERS signals of R6G in the regions marked with A, B, and C in (**a**) in comparison with those in Region II of the single-line ablated Si substrate.

Single-line laser ablation only created limited SERS-active area on the substrate surface, which was not convenient in practical detections. So large-area SERS-active substrates were fabricated by multi-line laser ablation. In order to further optimize the distribution of nanoparticles to promote the SERS performance, the distance between the center of adjacent microgrooves was set to 80 μm, making the regions with the best SERS performance coincident. Figure 3a shows the microscope image of multi-line laser-ablated Si surface. The center region of adjacent microgrooves marked with B is the coincident regions with the best SERS performance, appearing with a dark gray value. It is 20~40 μm away from the adjacent two microgrooves with twice depositions of nanoparticles. Its neighboring non-ablated regions are tagged by A and C, respectively. Figure 3b shows the SEM image of Region B. It exhibits quasi-3D structures with randomly arranged and different shaped nanoparticles aggregated in more than one layer, which are created by the double depositions of nanoparticles from the two adjacent microgrooves. The SERS signals were collected after 30 nm Ag film deposition followed by monolayer R6G molecules decoration. Figure 3c illustrates the SERS signals of R6G molecules absorbed on the multi-line ablated Si substrate in comparison with those from Region II of the single-line ablated Si substrate. Both the substrates were coated with 30 nm Ag film. The SERS signals of Region B have a 1.8-fold enhancement compared with Region II since more hotspots are created on the quasi-3D structures induced by the twice nanoparticles depositions, and meanwhile the quasi-3D morphology of Region B creates larger surface area to absorb more probe molecules.

### 3.2. SERS Performance of the G/Ag/LTSi Substrate

**Figure 4:**
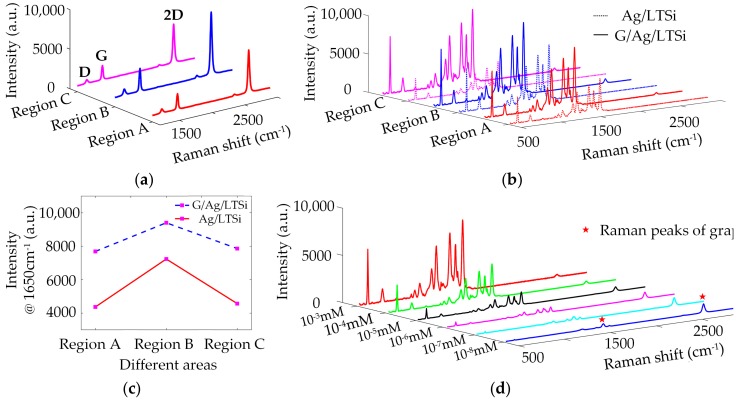
(**a**) Raman spectra of graphene on the G/Ag/LTSi substrate; (**b**) SERS signals; and (**c**) SERS intensity at 1650 cm^−1^ of R6G on the Ag/LTSi and G/Ag/LTSi substrates; (**d**) SERS signals of R6G with different concentrations from 10^−3^ to 10^−8^ mM absorbed on the G/Ag/LTSi substrates.

The Ag-based SERS substrates are easy to be oxidized when exposed to ambient conditions. The oxidation of Ag nanostructures will change their plasmonic property, resulting in the significant deterioration of SERS performance. To protect the Ag nanostructures from oxidation, a monolayer graphene serving as a protective layer was transferred to the surface of Ag/LTSi to form a large-area G/Ag/LTSi SERS substrate. Furthermore, the as-transferred monolayer graphene can provide an additional enhancement of Raman signals attributed to the CM and function as a spacer to avoid the direct contact of probe molecules with Ag nanostructures. The as-transferred monolayer graphene was synthesized by the copper-catalyzed CVD method and transferred via the PMMA-assisted wet chemical etching. Figure 4a shows the Raman spectra of graphene on the G/Ag/LTSi substrate. The relative intensity ratio of I_2D_ to I_G_ is 2.22–2.31, indicating monolayer feature of the as-transferred graphene. The intensity of graphene Raman signals in Region B is stronger than that in Regions A and C, which is due to better enhancement capacities for Raman signals in Region B. The covering graphene can be regarded as a probe molecule on the Ag/LTSi substrate herein.

Figure 4b illustrates the Raman spectra of 10^−2^ mM R6G absorbed on the G/Ag/LTSi substrate in comparison with those on the Ag/LTSi substrate without graphene covering. The regions with graphene covering exhibit stronger SERS signals than the same regions without graphene covering. This is owing to the additional chemical enhancement of SERS signals and the enrichment of R6G molecules contributed by the covering graphene layer. However, the enhancement of SERS signals provided by graphene is not as good as the graphene SERS substrate reported previously [17], where graphene affords an EF of 2–17. This is probably since the covering graphene layer is not completely contacted with the uneven surface morphology of underlying Ag nanostructures, leading to the increase of distance between the probe molecules and the Ag nanostructures in local areas. Figure 4c shows the SERS intensity at 1650 cm^−1^ from both the G/Ag/LTSi and Ag/LTSi substrates. The SERS signals are enhanced by 1.775 times and 1.73 times in the Regions A and C with graphene covering, which are greater than Region B with only a 1.31-time enhancement after graphene covering. Region B with quasi-3D surface structures exhibits a rougher surface morphology than Regions A and C. The less rough surface morphology of Regions A and C make the covering graphene layer have better contact with the Ag nanostructures. So that more probe molecules in Regions A and C are closer to the Ag nanostructures. The enhancement of electromagnetic field generated by the Ag nanostructures in Regions A and C can be better employed to boost the SERS signals.

The detection limit of G/Ag/LTSi substrate was investigated by detecting R6G solutions with different concentrations, as shown in Figure 4d. The intensities of SERS signals from R6G gradually decline with the decrease of R6G concentration. Some main Raman peaks of R6G, such as 612, 1360, 1509, and 1650 cm^−1^ can be detected from the G/Ag/LTSi substrate functionalized with 10^−7^ mM R6G solution, whereas Raman peaks of R6G cannot be observed almost from the collected SERS signal of 10^−8^ mM R6G solution. Therefore 10^−7^ mM can be regarded as the detection limit of G/Ag/LTSi substrates for sensing R6G molecules at the detection conditions with 10 mW laser power and two seconds of integration time. Besides the characteristic Raman peaks of R6G, some weak Raman peaks from graphene, marked by red asterisks, can be clearly observed, especially during detecting the low concentration R6G solutions. The SERS signals from graphene layer have a little growth with the decrease of R6G concentration. That is because only a few R6G molecules are absorbed on the G/Ag/LTSi substrate when detecting low-concentration R6G solution, which means only a fraction of the substrate’s surface is covered by R6G molecules. More graphene area without R6G covering can be irradiated by the focal laser spot to generate more Raman signals of graphene during detecting the SERS signals of R6G solution with lower concentration.

The EF of G/Ag/LTSi substrates can be estimated by Equation (1) [33].

(1) $$EF=\frac{I_{SERS}/N_{SERS}}{I_{Raman}/N_{Raman}}$$

where *I_SERS_* is the intensity of specified Raman peak from the probe molecules adsorbed on the G/Ag/LTSi SERS substrate. *I_Raman_* is the intensity from the normal Raman measurement without using SERS substrates. *N_SERS_* and *N_Raman_* are the numbers of molecules contributing to the SERS and normal Raman signals, respectively. Since the fluorescence generated by R6G molecules at the excitation wavelength of 532 nm is much stronger than Raman signals when using normal Raman measurement without SERS substrates or the addition of fluorescence quencher. It is difficult to obtain the exact Raman intensity of R6G molecules at the specified Raman peak. Therefore, 4-MBT molecules, another commonly used probe molecule without fluorescence at the 532 nm excitation wavelength, were utilized to calculate the EF. 4-MBT molecules have the characteristic Raman peaks at ~1083 cm^−1^ and ~1575 cm^−1^ derived from the in-plane ring-breathing mode coupled with the C-S mode and the C-C stretching mode [34]. Since the normal Raman signal intensity is intrinsically weak, the measurement of *I_Raman_* needs to be performed at much higher laser power and longer accumulation time. When calculating the EF, the measured *I_Raman_* should be normalized to the same excitation power and accumulation time with *I_SERS_* measurement. Utilizing 100 mM 4-MBT solution as probe molecules, the average *I_SERS_* on Region B is 13857 counts and the normalized *I_Raman_* is 0.0359 counts. *N_Raman_* can be calculated via Equation (2).

(2) $$N_{Raman}=\mathrm{NA}\times \rho \times h\times A$$

where NA is the Avogadro’s number, *ρ* is the concentration, h and A are the focal depth and area of laser spot respectively, which can be obtained by measuring the intensity profile of fluorescence beads in ethanol. The measured area and focal depth are ~0.81 μm^2^ and ~4.92 μm, respectively. In the ethanol solution of 4-MBT with a concentration of 100 mM, *N_Raman_* is approximately 2.39 × 10^8^ molecules. For *N_SERS_*, the 4-MBT surface density is assumed as 4.5 × 10^14^ molecules/cm^2^ [35]. There are approximately 3.645 × 10^6^ molecules located in the detection volume. The average enhancement factor is estimated to be ~2.6 × 10^7^ based on Equation (1).

### 3.3. Stability of the G/Ag/LTSi Substrate

**Figure 5:**
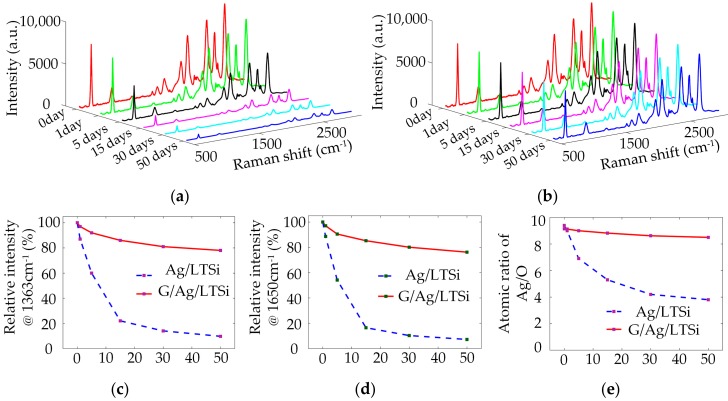
SERS signals of R6G from (**a**) the Ag/LTSi substrate and (**b**) the G/Ag/LTSi substrate when exposed to ambient air for 0, 1, 5, 15, 30, 50 days, respectively; the variation of normalized SERS signals at (**c**) 1363 cm^−1^ and (**d**) 1650 cm^−1^ from both the Ag/LTSi and G/Ag/LTSi substrates versus exposure time in ambient conditions; (**e**) atomic ratio of Ag/O versus exposure time in ambient conditions measured by EDS.

Stability is an important issue for SERS substrates in practical applications. The stability of G/Ag/LTSi substrates was investigated in this subsection. The G/Ag/LTSi and Ag/LTSi SERS substrates were prepared and exposed to ambient air at room temperature for 1, 5, 15, 30, and 50 days, respectively. Then the SERS signals of 0.1 mM R6G were collected from Region B of each substrate. Figure 5a,b show the evolutions of SERS signals on the G/Ag/LTSi and Ag/LTSi SERS substrates when exposed to ambient air. The variation of Raman intensities at 1363 cm^−1^ and 1650 cm^−1^ versus exposure days are plotted in Figure 5c,d, where intensities of SERS signals are normalized to those from the freshly prepared substrates for comparison. The SERS signals obtained from both the substrates decrease over exposure time. The SERS signals from Ag/LTSi substrate drop to 86.4% at 1363 cm^−1^ and 87.7% at 1650 cm^−1^ of the original intensity after only one day of exposure to ambient conditions, whereas the SERS signals from G/Ag/LTSi substrate remain almost unchanged. After a 50 days of exposure to ambient air, the SERS signals from Ag/LTSi substrate significantly decrease by 96.4% at 1363 cm^−1^ and 93.3% at 1650 cm^−1^. In comparison, the SERS signals from G/Ag/LTSi substrate decrease less than 23% both at 1363 cm^−1^ and 1650 cm^−1^. The G/Ag/LTSi substrate still maintains good SERS performance, even after 50 days of exposure to ambient air due to the protection of covering graphene.

To further confirm the anti-oxidation effectiveness provided by the covering monolayer graphene, EDS analysis was carried out to roughly reveal the changes of chemical composition on the substrate surfaces over exposure time. Figure 5e illustrates the ratios of Ag to O versus exposure time on both the substrates with and without graphene covering. The initial Ag/O ratios are 9.58 for the freshly prepared Ag/LTSi substrates and 9.23 for the G/Ag/LTSi substrates, respectively. The oxygen element of freshly prepared SERS substrates mainly comes from the oxidation of Si surface during laser ablation. The Ag/O ratio of Ag/LTSi substrate decreases significantly over exposure time. After a 50-day exposure to ambient conditions, the Ag/O ratio decreases to 3.81, which implies that the Ag nanostructures have been severely oxidized to cause an increase of oxygen content. Nevertheless, the Ag/O ratio of G/Ag/LTSi substrate only changes from 9.23 to 7.85 after a 50-day exposure, which is much more slowly than that of Ag/LTSi substrate. These results suggest that the G/Ag/LTSi substrate is much more stable than the Ag/LTSi substrate and the monolayer graphene of G/Ag/LTSi substrate can provide long-term and effective protection for the covered Ag nanostructures.

## 4. Conclusions

In conclusion, a stable and sensitive SERS substrate based on the novel hybrid structure of G/Ag/LTSi was developed and a simple, rapid, and low-cost fabrication approach was explored. Pulse laser ablation was employed to directly generate nanoparticles on the Si surface. The distribution of nanoparticles was investigated and optimized by tuning the distance of adjacent microgrooves created by multi-line laser ablation. The center area of adjacent microgrooves exhibits quasi-3D structures with abundant nanoparticles aggregated in more than one layer. With a 30 nm Ag film coating, substantial Ag nanostructures were formed and abundant hotspots were created for SERS detections. A CVD-grown monolayer graphene was transferred to the surface of Ag nanostructures as a protective layer to prevent the Ag nanostructures from oxidation. Moreover, the covering graphene also functions as an enhancer to boost the Raman signals ascribed to the CM. By the detection of R6G and 4-MBT molecules, the G/Ag/LTSi substrate manifests a sensitive SERS performance with a detection limit of 10^−7^ mM and an enhancement factor over 10^7^. Furthermore, the G/Ag/LTSi substrate exhibits a better stability than the Ag/LTSi substrate since the graphene layer prevents the direct contact of Ag nanostructures with air and water vapor. Less than 23% SERS signals lost when the G/Ag/LTSi substrate were exposed to ambient conditions for 50 days. For the present G/Ag/LTSi substrate, the covering graphene layer plays crucial roles in the protection of Ag nanostructures and the enhancement of Raman signals. The present strategy in this work is a useful and convenient route to prepare robust and sensitive substrates for SERS detection.
